# The Interference Mechanism of Basil Essential Oil on the Cell Membrane Barrier and Respiratory Metabolism of *Listeria monocytogenes*

**DOI:** 10.3389/fmicb.2022.855905

**Published:** 2022-04-01

**Authors:** Changzhu Li, Chenghui Zhang, Xiaochen Chen, Haiying Cui, Lin Lin

**Affiliations:** ^1^State Key Laboratory of Utilization of Woody Oil Resource, Hunan Academy of Forestry, Changsha, China; ^2^School of Food and Biological Engineering, Jiangsu University, Zhenjiang, China

**Keywords:** basil essential oil, anti-*Listeria monocytogenes* mechanism, cell membrane barrier, respiratory metabolism, molecular docking

## Abstract

In order to prevent food-borne diseases caused by *Listeria monocytogenes* (*L. monocytogenes*) safely and effectively, plant essential oils that have no toxic side effects and are not prone to drug resistance have become the focus of research. This article takes basil (*Ocimum basilicum* L.) essential oil (BEO) as the research object and explores its antibacterial mechanism against *L. monocytogenes*. The site of action was preliminarily determined to provide a theoretical basis for the development of natural antibacterial agents. The results show that BEO has good antibacterial activity against *L. monocytogenes*. After 8 h of treatment with BEO (1 mg/ml), the number of remaining bacteria reached an undetectable level. Combining spectroscopic analysis techniques (Raman, UV, and fluorescence spectroscopy) and fluorescence microscopy imaging techniques, it was found that BEO increased the disorder of the hydrocarbyl chain of phospholipid tail, which in turn led to increased cell membrane permeability, thereby causing the leakage of intracellular proteins and DNA. Meanwhile, respiratory metabolism experiments showed that BEO inhibited the EMP pathway by inhibiting the activity of key enzymes. From the molecular docking results, this inhibition may be attributed to the hydrophobic interaction between α-bergamotene and the amino acid residues of phosphofructokinase (PFK) and pyruvate kinase (PK). In addition, BEO can also cause oxidative stress, and reactive oxygen species (ROS) may also be related to the damage of cell membranes and enzymes related to respiratory metabolism.

## Introduction

According to a statistical report from the WHO, 600 million people worldwide are infected with food-borne diseases, and 420,000 deaths are caused each year (World Health Organization, 2020). The economic loss caused by this in developing countries is as high as 110 billion US dollars each year (Zamuz et al., 2021). Listeriosis is a zoonotic disease caused by *Listeria monocytogenes*. *Listeria monocytogenes* is highly adaptable to the external environment and can survive in low temperatures, high salt, and a wide pH range (Nyhan et al., 2021), so it can contaminate most foods. After people ingest foods infected by *L. monocytogenes*, they will experience varying degrees of symptoms and even death. Common symptoms of infection include headache, diarrhea, vomiting, etc. However, severe symptoms, such as meningitis, sepsis, and miscarriage, may occur in people with weakened immune systems (pregnant women, infants, and the elderly). Although there are few cases of listeriosis, the fatality rate is high (20–30%), and it is considered a significant public health problem (Cui et al., 2018). From 2009 to 2015, *L. monocytogenes* was the leading cause of death from food-borne pathogens in the United States, causing 52% of epidemic-related deaths (Huang et al., 2021). In the past few years, the confirmed cases of listeriosis in EU statistics have shown a significant increase (EFSA, 2019). It can be seen that *L. monocytogenes* contamination has caused a significant social and economic burden on the public health of the world. Therefore, it is very urgent to find a safe and effective method to control *L. monocytogenes*.

Unlike chemically synthesized antibacterial agents, natural antibacterial agents are extracted and purified from natural raw materials and have the advantages of safety, no toxic side effects, etc. As an important source of natural antibacterial agents, essential oils of plant-derived secondary metabolites have recently become the subject of many studies. Essential oils are composed of various active small molecules, which have broad-spectrum antibacterial activity and are not prone to drug resistance (Kalily et al., 2016). Therefore, it is a potential substitute for chemically synthesized antibacterial agents. It has considerable application prospects and economic value in industries, such as food preservation, biomedicine, and cosmetics.

Basil (*Ocimum basilicum* L.) is an herb, mainly used as a condiment or spice, and also used in the food, perfume, and pharmaceutical industries (Kordi et al., 2020). Its wide application is attributed to the rich secondary metabolites of the basil species, and the basil essential oil (BEO) is its primary functional activity substance. BEO has been found to have the properties of killing mosquitoes, antibacterial, insecticidal, antifungal, and antioxidant properties, among which antibacterial activity has been extensively studied (Amor et al., 2021). Studies have found that BEO has antibacterial activity against most Gram-positive bacteria, Gram-negative bacteria, and pathogenic fungi. However, research on antibacterial mechanism of BEO is minimal. At present, the research on the antibacterial mechanism of essential oils mainly focuses on cell membrane barrier, intracellular environment, and physiological metabolism, etc.

The cell wall and cell membrane are the main cell structures of bacteria, which play the role of information transmission, energy conversion, and material transportation, and are the first line of defense for bacterial cells against harsh environments (Lee et al., 2013; Henrik and Jeff, 2017). Therefore, the cell barrier is considered the primary and critical target of EOs. These fat-soluble plant essential oils are widely believed to have an irreversible effect on the structure of cell membranes. When essential oils penetrate into cells, they will act on intracellular macromolecular substances and eventually interfere with physiological metabolism. Respiratory metabolism is the primary way for most bacteria to produce energy (Dai et al., 2020). Therefore, this study reveals the antibacterial mechanism of BEO against *L. monocytogenes* from three aspects: cell membrane barrier, metabolism (respiratory metabolism), and genetic material. Moreover, the specific targets of BEO on cell membranes, respiratory metabolism, and DNA were analyzed through hydrogen nuclear magnetic resonance (NMR) spectroscopy and molecular docking technology.

## Materials and Methods

### Bacterial Strains and Culture

*Listeria monocytogenes* EDG-e strain was purchased from Beina Chuanglian Biotechnology Research Institute (Beijing, China). The strain was inoculated in Peptone Yeast Glucose (PYG) medium at 37°C for 48 h. Basil (*Ocimum basilicum* L.) essential oil was bought from JE International (Caussols, France). The Taxonomic Serial number for *Ocimum basilicum* L. is 32,627. BCA kit was purchased from Nanjing Jiancheng Bioengineering Research Institute Co., Ltd. (China). HK, PFK, and PK kits were purchased from Suzhou Keming Biotechnology Co., Ltd. (China). The chemical reagents used in the experiments were purchased from Sinopharm Chemical Reagent Co., Ltd (China).

### Chemical Composition of BEO

The GC/MS device (Agilent 6890GC/5973NMSD, Agilent Technologies, United States) is used to detect the composition of BEO. The injection temperature was 250°C in the test. The temperature of chromatographic column is set to 60°C (2 min), and then temperature is increased to 230°C (5 min) at a rate of 4°C/min. The component identification of BEO is based on mass spectrometry and retention time compared with standard library. The relative concentration of the compound is determined by area normalization.

### Antibacterial Activity of BEO Against *Listeria monocytogenes*

The broth microdilution method was used to determine the minimum inhibitory concentration (MIC) and minimum bactericidal concentration (MBC) of BEO (Andrews, 2001). The bactericidal curve of BEO against *L. monocytogenes* was assessed by plate count method and a time–kill curve was drawn (Zhou et al., 2021). BEO was dissolved in a small amount of absolute ethanol and subsequently diluted to a fixed concentration with sterile PBS. Next, BEO was added to the bacterial suspension (1/2 MIC, MIC, and MBC), and the remaining bacterial counts were measured at 0.5, 1, 2, 4, and 8 h, respectively. The effect of BEO on the microstructure of the bacteria was observed by TEM (JSM-7001F, JEOL, Tokyo, Japan). The bacterial suspension was centrifuged (6,000 *g*, 10 min), washed, and resuspended in PBS, and then, BEO (MIC, MBC) was added for 4 h. After the reaction, the bacterial solution was dropped on the copper mesh, and 3% (w:w) phosphotungstic acid was added dropwise to stain for 3 min in the dark and then detected by TEM. Samples not treated with BEO were used as controls. The control group was added with the same amount of absolute ethanol as the experimental group.

### Effect of BEO on the Cell Membrane of *Listeria monocytogenes*

#### Effect of BEO on Cell Membrane Permeability of *Listeria monocytogenes*

The bacterial suspension cultured to the logarithmic phase was centrifuged (6,000 *g*, 10 min) to wash. The pellet was resuspended in 4 ml PBS, and different concentrations of BEO (1/2MIC, MIC, and MBC) were added to react for 4 h. After the reaction, 20 μl of Propidium iodide (PI) solution (20 mmol/L) was added to the sample and left in the dark for 30 min. Fluorescence images of different samples were obtained by an inverted fluorescence microscope (CX33, Olympus, Japan). The excitation wavelength and emission wavelength were set to 400 and 615 nm, respectively. In addition, changes in intracellular protein content, β-galactosidase, and electrical conductivity can also reflect the effect of BEO on cell membrane permeability. Protein leakage is detected using BCA protein detection kit (Kang and Song, 2019). Different concentrations of BEO were added to the bacterial suspension cultured to the logarithmic phase to react for 4 h. Subsequently, the suspension was centrifuged, and the supernatant was diluted 10 times to detect the changes in the conductivity of different samples. BEO (MIC, MBC) and 100 μl of 1 mg/ml ONPG (O-Nitrophenyl β-D-galactopyranoside) were added to 1 ml of bacterial solution. The mixed solution was incubated at 37°C for 4 h, and then the OD_420nm_ value was measured. The control group is the sample that has not been treated with BEO.

#### Analysis of the Site of Interaction Between BEO and Cell Membrane

##### Determination of the Interaction Between BEO and Cell Membrane

The *L. monocytogenes* suspension (10^5^–10^6^ CFU/ml) was treated with BEO (MIC, MBC) for 4 h, followed by centrifugation (6,000 *g*, 10 min) to obtain the cell pellet and washed. Subsequently, the bacterial pellet was collected and treated with a freeze-drying apparatus for 48 h, and the bacterial powder was obtained for use. The lyophilized bacterial powder (200 mg) was placed in a beaker, and 35 ml of the mixed solution (V_methanol_: V_chloroform_ = 1:2) was added (Huang and Kim, 2016). After mixing, the samples were sonicated for 30 min. Next, the samples were magnetically stirred for 2 h and then centrifuged to separate the phases (2,000 rpm, 20 min). The supernatant is the phospholipid phase. The phospholipid phase was filtered through a 0.45-μm filter membrane, and the filtrate was blown with nitrogen to obtain cell membrane phospholipids. A small number of phospholipids are placed under a Raman microscope (SR550BR, Pu Shi Nano Technology Co., Ltd., Xiamen, China) to measure the spectrum. The excitation wavelength is set to 785 nm, and the scanning range is 1,000–1,200 cm^−1^.

##### 1^H^ HNM Analysis of BEO Action Site

The cell membrane phospholipids were extracted according to the method described in section “Determination of the Interaction Between BEO and Cell Membrane.” Since the main component of BEO is linalool, so linalool was chosen to study the possible action sites of BEO and cell membrane. The extracted phospholipids were dissolved in a chloroform solution, and different concentrations of linalool were added to react for 3 h. After the reaction, it was placed in a nitrogen-blowing apparatus until the solvent was completely volatilized. Deuterated methanol was used as the solvent, and the samples were analyzed by 1^H^ NMR (AVANCEII 400 MHz, BRUKER, Germany).

### Oxidative Stress Response of *Listeria monocytogenes* Cells

2′,7′-Dichlorofluorescein yellow diacetate (DCFH-DA) was used to detect intracellular reactive oxygen species (ROS). The bacterial suspension (4 ml) in the logarithmic growth phase was centrifuged (8,000 *g*, 10 min), washed, and the bacterial pellet was collected. The bacterial pellet was resuspended in 1 ml of DCFH-DA (10 μmol/L) solution and reacted in the dark for 30 min. BEO (MIC) concentration was added to the sample and reacted in the dark for different times (30, 45, and 60 min). After the reaction, the sample was centrifuged (8,000 *g*, 10 min), washed, and then resuspended in 1 ml of PBS (Yang et al., 2020). The control group was a sample that was not treated with BEO. An inverted fluorescence microscope was used to detect the production of reactive oxygen species in the cells. At the same time, the fluorescence intensity of different samples is measured by a fluorescence spectrophotometer (F-4500, Hitachi, Japan). The excitation wavelength and emission wavelength are 488 and 525 nm, respectively.

### Effect of BEO on Respiratory Metabolism of *Listeria monocytogenes*

#### Determination of the Main Inhibitory Pathways of BEO on the Respiratory Metabolism

The typical inhibitor inhibition method was used to analyze the influence of BEO on the respiratory metabolism pathway of *L. monocytogenes*. PBS (3.6 ml, 0.1 M, pH 7.0), glucose solution (0.4 ml, 1%), and *L. monocytogenes* suspension (1 ml, 10^6^ CFU/ml) were added to the beaker, exposed to air and stirred for 5 min, then closed beaker. Subsequently, a dissolved oxygen meter was used to measure dissolved oxygen and obtain the initial respiration rate R_0_. Iodoacetic acid, oxalic acid, sodium phosphate (500 mg/L), and BEO (MIC) were added to the initial system respectively. The respiratory rate R_1_ of each group was calculated, and the respiratory inhibition rate (I_R_) was calculated. Then, three inhibitors were added to the system containing BEO respectively, the respiratory rate R_2_ was measured, and the respiratory superposition rate (S_R_) was calculated (Lin et al., 2018).

#### Molecular Docking

The Dock 6.9 software realizes the molecular docking of phosphofructokinase (PFK), pyruvate kinase (PK), and linalool. The amino acid sequence of the acceptor molecule is from the NCBI database [the amino acid sequence of *L. monocytogenes* hexokinase (HK) is unknown]. Swiss Model Workspace^1^ is used to construct the three-dimensional structure of the protein, from which the appropriate template is selected and the crystal structure is downloaded from the PDB protein data (Phosphofructokinase PDB ID: 6pfk, Pyruvate kinase PDB ID: 2e28).^2^ The structures of linalool, cineole, and α-bergamotene are from PubChem database.^3^ Then, the Yinfo Technology Cloud Computing Platform^4^ was used to process the PDB structure of the receptor, including adding hydrogen atoms, separating receptors and ligands, deleting redundant chains, and removing water molecules. At the same time, the ligand is hydrogenated, charged, and protonated. The active site of pyruvate kinase was prepared according to the study of El Sayed et al. (2020). The platform is used to identify the binding site of phosphofructokinase. Dock 6.9 was used for docking of flexible ligands and analysis of the results.

#### The Effect of BEO on the Activity of Key Enzymes in Respiratory Metabolism

The effects of BEO on the enzymatic activities of HK, PFK, and PK were determined by the corresponding HK, PFK, and PK kits.

### Statistical Analysis

All the above experiments were repeated three times, and the average value was taken. Error bars are expressed as mean ± SD. The same letter in the figure indicates no significant difference (*p* < 0.05). The experimental results were analyzed by SPSS 24.0 software (IBM Corp., Armonk, NY) and Origin 2020.

## Result and Discussion

### Chemical Composition of CEO

**Figure 1:**
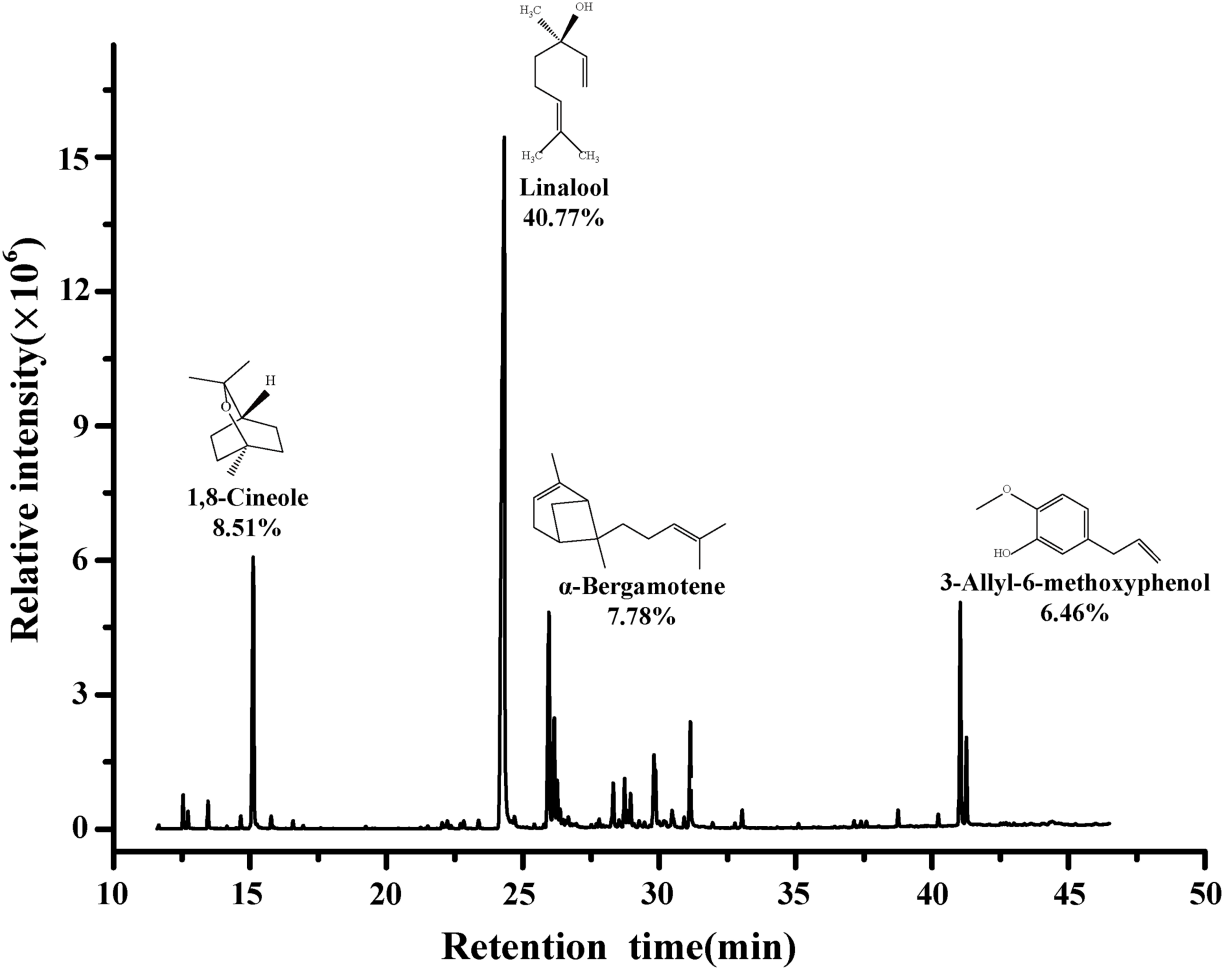
Chemical composition of BEO.

The main composition of BEO determined by GC–MS are shown in Figure 1. The composition of BEO includes linalool (40.77%), 1,8-cineole (8.51%), α-bergamotene (7.78%), 3-allyl-6-methoxyphenol (6.46%), γ-cadinene (3.59%), β-elemene (3.33%), and other compounds. This result is similar to that of Milenković et al. (2019). So, the main constituents of BEO are terpenes, which have been proven to have broad-spectrum antibacterial activity. Linalool, which accounts for the highest proportion of BEO, belongs to the chain terpene alcohols, which has higher antibacterial activity (He et al., 2022), but the activity is lower than BEO, indicating that the antibacterial activity of BEO comes from the synergistic effect of multiple components.

### Antibacterial Activity of BEO

**Figure 2:**
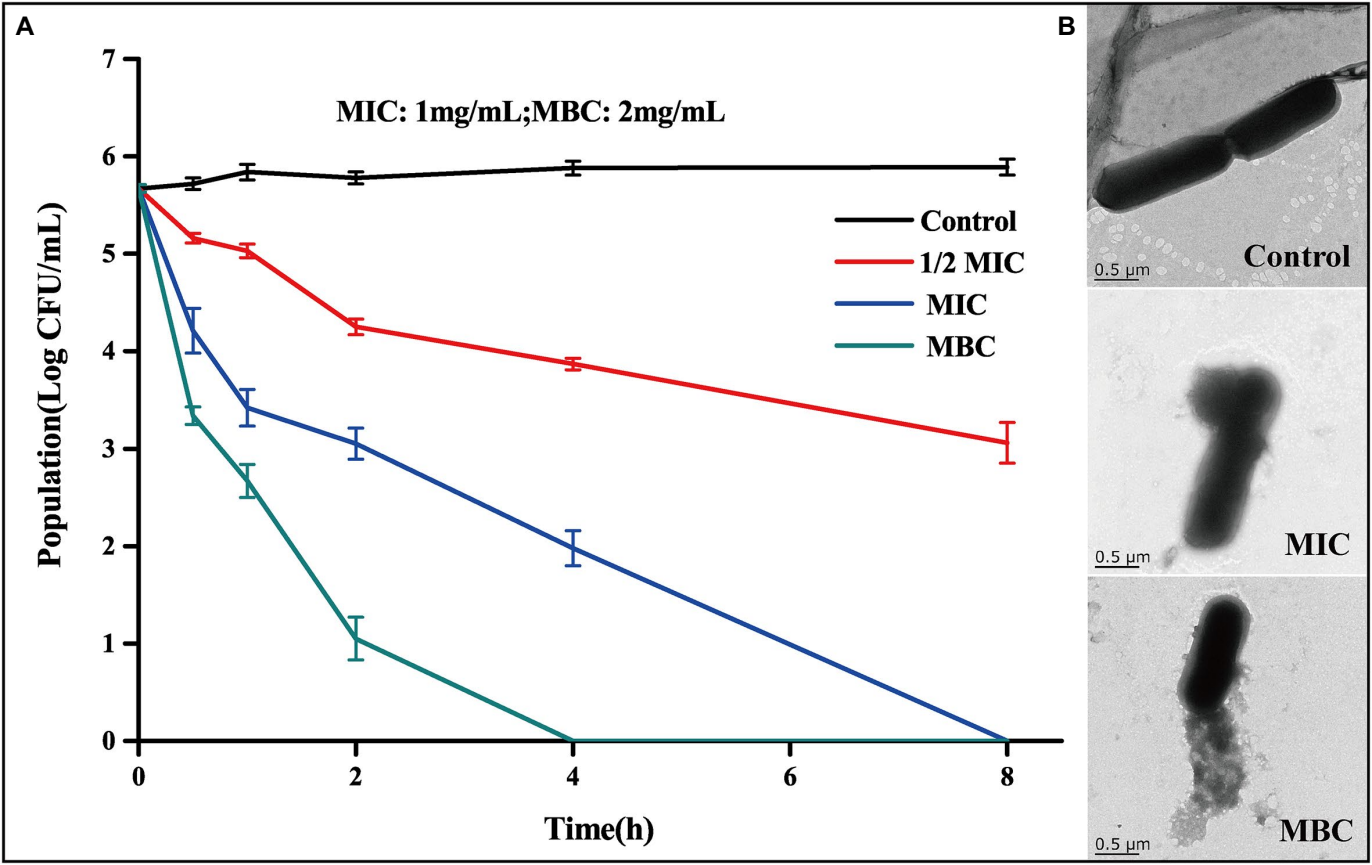
Time kill curve of BEO against *Listeria monocytogenes* **(A)**; TEM images of *L. monocytogenes* before and after BEO [minimum inhibitory concentration (MIC), minimum bactericidal concentration (MBC)] treatment **(B)**.

The measured MIC and MBC of BEO against *L. monocytogenes* were 1 mg/ml and 2 mg/ml, respectively. The antibacterial activity of BEO was evaluated by a bactericidal curve (Figure 2A). The amount of *L. monocytogenes* in the control group was maintained at about 5.7 log CFU/ml. Compared with the control group, the addition of BEO caused a significant change in the number of *L. monocytogenes*. The 1/2 MIC of BEO showed that the sterilization rate reached 99.75%. The number of *L. monocytogenes* had reached an undetected level at 8 h when concentration of BEO raised to MIC. What is more, the number of remaining bacteria reaches the undetected level within 4 h when concentration of BEO is MBC. The above results indicate that BEO has an excellent inhibitory effect on *L. monocytogenes* and can be used as a natural antibacterial agent.

Studies have shown that one of the main targets of essential oils on bacteria is the cell wall and cell membrane, which can cause irreversible damage to the cell barrier (Hyldgaard et al., 2012). TEM reflects the changes in the morphology of *L. monocytogenes* cells after the action of BEO (Figure 2B). The cell structure of the control group is complete, showing a full and round rod-shaped structure. After treatment with MIC and MBC concentrations of BEO, cell structure was significantly damaged, cell integrity was destroyed, intracellular material leaked, and cell morphology shrank. This effect may be mainly due to the presence of a variety of strong fat-soluble terpenes in BEO, such as linalool, 1,8-cineole, and α-bergamotene. These substances have been proven to dissolve components of bacterial cell walls and interact with cell membranes (Sipponen et al., 2009). The above results indicate that BEO irreversibly damages the cell structure of *L. monocytogenes*.

### The Effect of BEO on the Cell Membrane of *Listeria monocytogenes*

#### Effect of BEO on Cell Membrane Permeability

From the above, BEO will cause cell structure changes, which may increase the permeability of the cell membrane, and further cause the leakage of intracellular DNA, proteins, intracellular ions, and other macromolecular substances. Therefore, the changes in cell membrane permeability after effect of BEO were measured.

**Figure 3:**
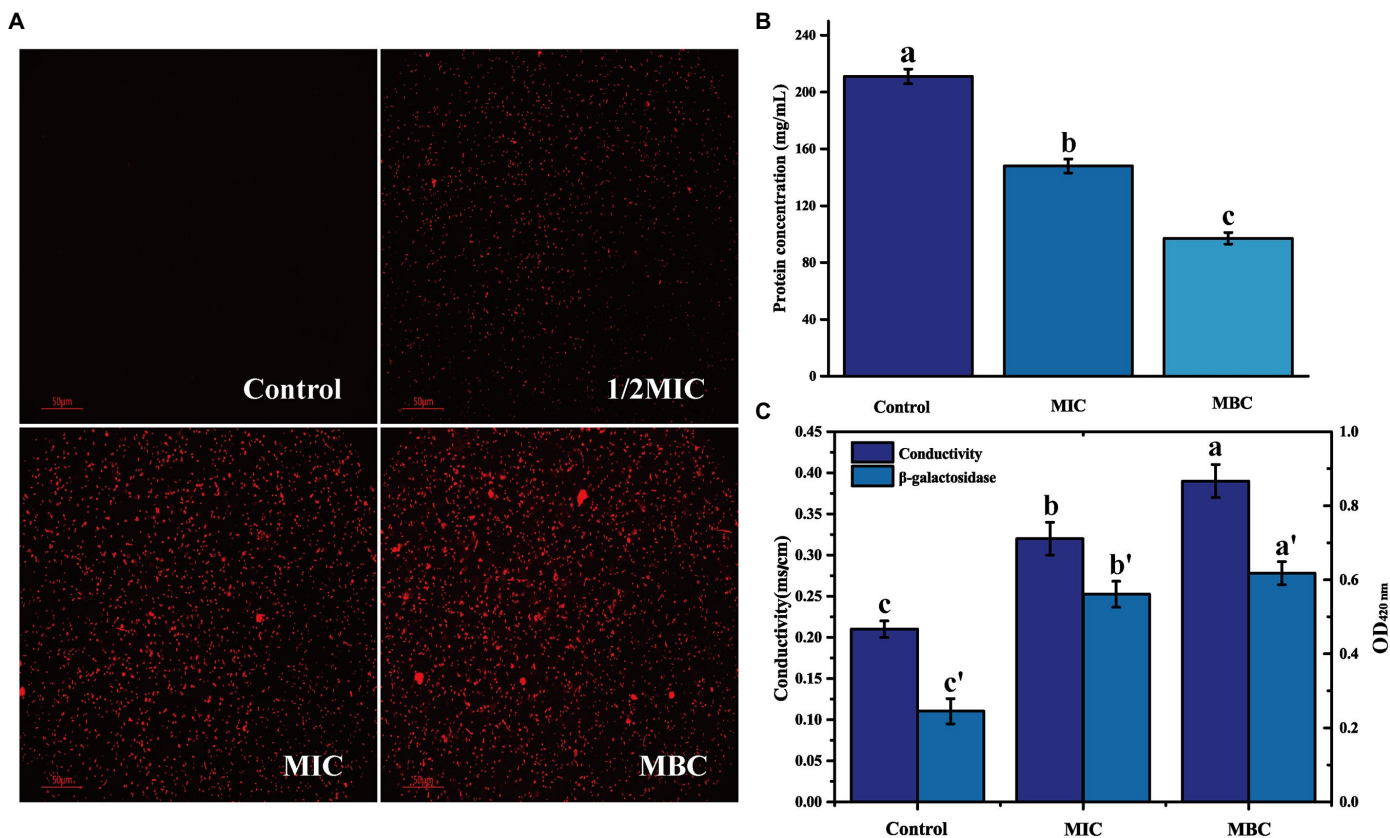
The effect of BEO on the cell membrane permeability of *L. monocytogenes*: **(A)** Propidium iodide (PI) fluorescence picture; **(B)** Intracellular protein content; and **(C)** Changes of electrical conductivity and β-galactosidase. Different letters in the figure indicate significant difference ( *p* <0.05).

Propidium iodide is a membrane-impermeable fluorescent probe. In general, it cannot penetrate the complete cell membrane. When the permeability of the cell membrane increases, PI can penetrate the cell membrane and combine with genetic material to stimulate red fluorescence (Davey and Hexley, 2011). There is no red fluorescence in the control group, indicating that the *L. monocytogenes* cell membrane is intact (Figure 3A). After treatment with 1/2MIC, MIC, and MBC concentration of BEO, a large amount of red fluorescence appeared in the samples. This red fluorescence indicated that BEO caused an increase in cell membrane permeability, which facilitated the penetration of a large amount of PI into *L. monocytogenes* cells, where it binds to DNA and eventually emits red fluorescence. The leakage of intracellular protein can indicate changes in cell membrane permeability. As shown in Figure 3B, compared with the control group, the soluble protein content of the MIC and MBC experimental groups decreased, indicating that BEO caused intracellular protein leakage. The soluble protein content in the control group was 211 mg/ml. After BEO treatment (MIC, MBC), the soluble protein content was 148 mg/ml and 97 mg/ml, which were reduced by 29.86 and 54.03%, respectively, indicating that BEO leads to an increase in cell membrane permeability. Changes in conductivity and β-galactosidase activity demonstrated the same results (Figure 3C). The conductivity of the control group was 0.21 mS/cm, while the conductivity of the MIC and MBC treatment groups was 0.32 and 0.39 mS/cm. The increase in cell membrane permeability will promote the leakage of intracellular ions and electrolytes, which will lead to an increase in electrical conductivity (Zhang et al., 2016). β-galactosidase is a crucial enzyme for normal physiological metabolism of bacteria, which can decompose lactose into galactose and glucose. ONPG is a lactose analog, which can be hydrolyzed by β-galactosidase into galactose and O-nitrophenol, the latter has an absorption peak at 420 nm ultraviolet wavelength (Song et al., 2012). The entry of ONPG into cells requires transport by lactose permease. Therefore, under normal circumstances, ONPG penetrates the cell membrane very slowly, but when the permeability of the cell membrane increases or even the membrane is damaged, ONPG will penetrate the cell membrane, and β-galactosidase will leak in a large amount. Overall this will lead to an increase in the amount of contact and reaction between the two, ultimately resulting in increased UV absorption at 420 nm. The OD_420nm_ value of the control group was 0.245, which was lower than 0.561 of the MIC treatment group and 0.618 of the MBC treatment group, indicating that the permeability of the cell membrane of *L. monocytogenes* increased after BEO treatment.

In short, the treatment of BEO will damage the cell membrane structure of the bacteria, increase the permeability of the cell membrane and cause the leakage of intracellular macromolecular substances and intracellular ionic electrolytes.

#### Analysis of the Site of Interaction Between BEO and Cell Membrane

##### Determination of the Interaction Between BEO and Cell Membrane

Raman spectroscopy is a powerful tool for studying changes in cell membrane structure and conformation (Lee and Bain, 2005). On the basis that BEO can cause damage to the cell structure of *L. monocytogenes*, Raman spectroscopy was used to analyze its interaction with the cell membrane. In the analysis of conformational changes in Raman spectroscopy, the study of phospholipids usually focuses on C-C tensile vibration and C-H tensile vibration. These vibration modes can get the same information, reflecting the degree of order or disorder of the lipid chain. Therefore, the interaction mode between BEO and phospholipids is analyzed using the C-C vibration mode.

**Figure 4:**
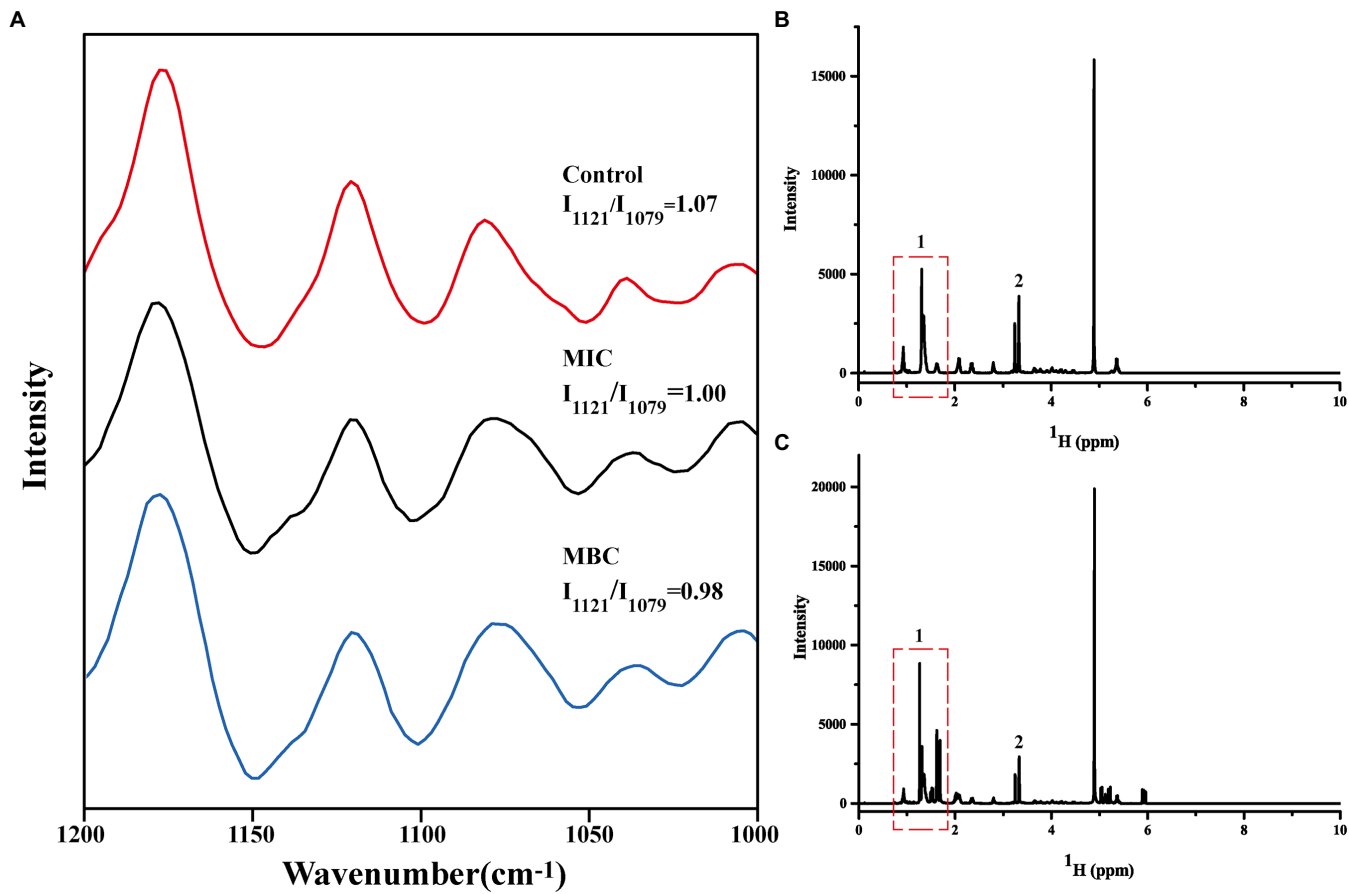
Changes in Raman spectra of cell membrane phospholipids **(A)**; One-dimensional ^1^H NMR spectra of cell membrane phospholipids before **(B)** and after **(C)** reaction with linalool.

First, the extracted phospholipids were analyzed by FTIR (Supplementary Figure S1). In the spectrum, the characteristic peak at 1,732 cm^−1^ is attributed to the stretching vibration of the C=O. The characteristic peaks at 2,863 cm^−1^, 2,926 cm^−1^, and 3,015 cm^−1^ represent the asymmetric and symmetric stretching vibrations of -CH_2_, and the asymmetric stretching vibrations of -CH_3_, respectively. The characteristic peak at 1,249 cm^−1^ is the result of the asymmetric contraction of -PO^−2^. The above results indicate that the phospholipids in the cell membrane have been successfully extracted. Then, the Raman spectrum of cell membrane phospholipids is shown in Figure 4A. The peaks between 1,070 and 1,130 cm^−1^ reflect the C-C vibration mode of phospholipids. The absorption peak at 1,079 cm^−1^ characterizes the twisted and rotated C-C conformation in the phospholipid fatty acid chain, while the absorption peak at 1,121 cm^−1^ describes the all-trans conformation of C-C (Petruk et al., 2013). The ratio of intensity of absorption peak at 1,121 cm^−1^ to intensity of absorption peak at 1,079 cm^−1^ (I_1121_/I_1079_) reflects the ratio of all-trans conformation and twisted and rotated conformation. The smaller the value, the greater the proportion of C-C twisted and rotated conformations in the cell membrane phospholipids, and the more disordered phospholipid fatty acid chain. The I_1121_/I_1079_ values of the control group, MIC, and MBC groups were 1.07, 1.00, and 0.98 in turn, indicating that BEO can interact with the phospholipid bilayer of *L. monocytogenes* and destroy the order of fatty acyl chain. It is speculated that this result is due to the top two components in BEO, linalool, and 1,8 cineole. Both are strongly lipid-soluble substances that can interact with the hydrophobic tails of phospholipids to disrupt membrane structure (Nguyen et al., 2017; Sun et al., 2018). Considering that the content of linalool is four times that of 1,8 cineole, and its activity is higher than that of 1,8 cineole, linalool was used for experiments in the subsequent exploration of action sites.

##### 1^H^ HNM Analysis of BEO Action Site

Nuclear magnetic resonance spectroscopy is often used for the structural analysis of organic matter (Jurczak et al., 2020). It is the most effective method to study the phase transition and structural changes of cell membranes, and it is also a powerful method to study the interaction of drugs with cell membranes. Therefore, we used ^1^H NMR to verify the interaction site of linalool with the cell membrane of *L. monocytogenes*.

It is well known that phospholipid molecules consist of water-soluble phosphate ends and water-insoluble hydrocarbon chain ends. The one-dimensional ^1^H spectrum of cell membrane phospholipid is shown in Figure 4B. The narrow linewidth and no peak split indicate that the prepared cell membrane phospholipid molecules are relatively uniform. The multiplet at the 0.8–1.9 ppm position (position 1) in the spectrum represents the hydrogen at the hydrocarbon chain end of the phospholipid. The resonance peak between 3 and 3.5 ppm represents the hydrogen of the phospholipid head (position 2). Figure 4C shows the spectrum of cell membrane phospholipids reacted with linalool (1 mg/ml). Compared with the control group, the multiplet at position 1 changed significantly, indicating that the hydrocarbyl chain changed significantly after the addition of linalool. This result suggests that linalool may act on the hydrocarbyl chains of the cell membrane of *L. monocytogenes*. Combining the results of Raman spectroscopy and ^1^H NMR, it can be concluded that BEO may affect the order of the phospholipid by acting on the hydrocarbyl chains of the phospholipid tail, thereby affecting function and physiological activity of cell membrane.

### Analysis of Oxidative Stress in *Listeria monocytogenes* Cells

In general, the production and removal of ROS in cells is a dynamic equilibrium process, and the cells are in a state of redox equilibrium at this time. When cells are exposed to extreme external environments, such as irradiation, antibacterial treatment, and ultraviolet radiation, the oxidative stress response in the cell is activated, and the redox balance is broken, resulting in the production of a large amount of intracellular ROS, which will attack the cell’s macromolecular substances and cell membrane from the inside, thereby accelerating cell death (Nogueira and Hay, 2013). The effect of BEO on the oxidative stress response of *L. monocytogenes* can be verified by the fluorescence changes of reactive oxygen species in the cell.

**Figure 5:**
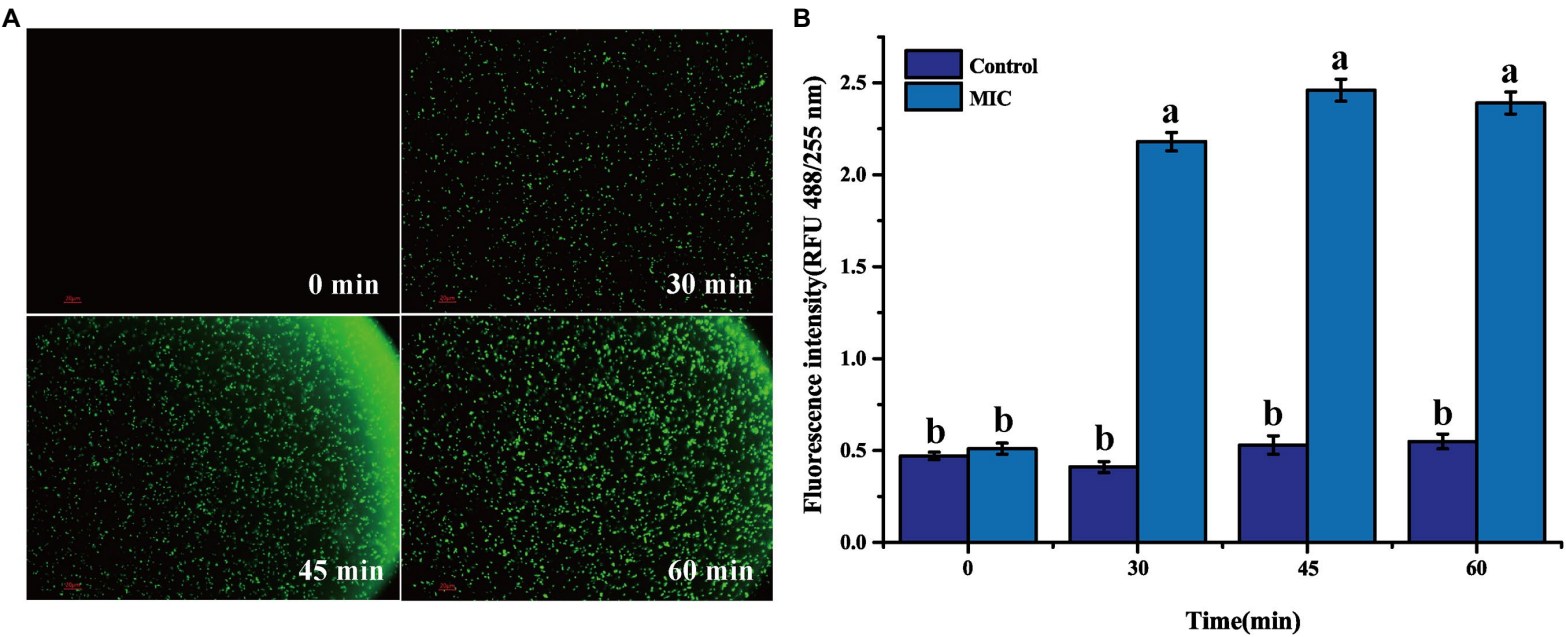
Reactive oxygen species (ROS) fluorescence picture of *L. monocytogenes* **(A)**; ROS fluorescence intensity **(B)**. Different letters in the figure indicate significant difference ( *p* <0.05).

2′,7′-Dichlorofluorescein yellow diacetate is used to detect the production of reactive oxygen species in cells. Under ROS action, DCFH-DA can generate 2′,7′-dichlorofluorescein (DCF) and produce green fluorescence (Zheng et al., 2019). After BEO (MIC) processes the *L. monocytogenes* for different times, a large amount of green fluorescence is gradually produced (Figure 5A). This green fluorescence indicates that BEO activates the internal oxidative stress response of *L. monocytogenes*, prompting it to produce a large amount of ROS. The same result was obtained by the kit determination. The fluorescence intensity of the control group remained unchanged within 60 min. In contrast, after BEO (MIC) treatment, the fluorescence intensity increased from 0.51 to 2.39 (Figure 5B), indicating that BEO stimulated *L. monocytogenes* produced a large amount of ROS.

### Effect of BEO on Respiratory Metabolism of *Listeria monocytogenes*

#### Determination of the Main Inhibitory Pathways of BEO on the Respiratory Metabolism

Respiratory metabolism in organisms refers to the oxidative metabolism of sugars in cells, which is the most important way to produce energy. Through respiration metabolism, bacterial cells obtain energy required for life activities and synthesize nutrients (such as protein and carbohydrates) needed for growth (Chen et al., 2020). Therefore, once the cell’s respiratory metabolism is inhibited, the cell’s normal physiological metabolism must be affected and cause the death of bacterial cells. There are three main respiratory metabolic pathways in organisms, namely, glycolysis pathway (EMP), pentose phosphate pathway (HMP), and tricarboxylic acid (TCA) cycle.

The typical inhibitor superimposed method can be used to determine the metabolic pathways that BEO mainly inhibits. Generally, the smaller the S_R_, the weaker the synergistic effect between BEO and typical inhibitors, indicating that the respiratory metabolism pathway inhibited by it may be the same as that of typical inhibitors. Both the typical inhibitor and BEO have an inhibitory effect on the respiratory metabolism of *L. monocytogenes* by measuring the respiratory inhibition rate (I_R_), and the inhibition rate after BEO treatment reached 34.69% (Table 1). On this basis, the S_R_ in Table 2 shows that the S_R_ of BEO and iodoacetic acid is the lowest, which is 9.52%. The metabolic pathway corresponding to iodoacetic acid is the EMP pathway, so the main inhibitory pathway of BEO on the respiratory metabolism of *L. monocytogenes* is the EMP pathway (Supplementary Figure S2). At the same time, it is speculated that the inhibitory effect of BEO on the EMP pathway is achieved by interacting with key regulatory enzymes in the pathway. When the EMP pathway is inhibited, the content of pyruvate entering the TCA cycle is reduced, and subsequent energy supply cannot guarantee physiological metabolic activities, causing metabolic disorders.

**Table 1:** The respiratory inhibitory rate of basil essential oil (BEO) and typical inhibitors.

Inhibitors	R_0_/μmol O_2_ (g.min)^−1^	R_1_/μmol O_2_ (g.min)^−1^	I_R_%
Iodoacetic acid	0.56	0.42	25.00
Malonic acid	0.68	0.50	26.47
Sodium	0.52	0.45	13.46
BEO	0.49	0.32	34.69

**Table 2:** The superposition rate (S_R_) of BEO and typical inhibitors.

Inhibitors	R_2_/μmol O_2_ (g.min)^−1^	S_R_ (%)
Iodoacetic acid + BEO	0.38	9.52
Malonic acid + BEO	0.36	28.00
Sodium phosphate + BEO	0.34	24.44

#### Molecular Docking

**Figure 6:**
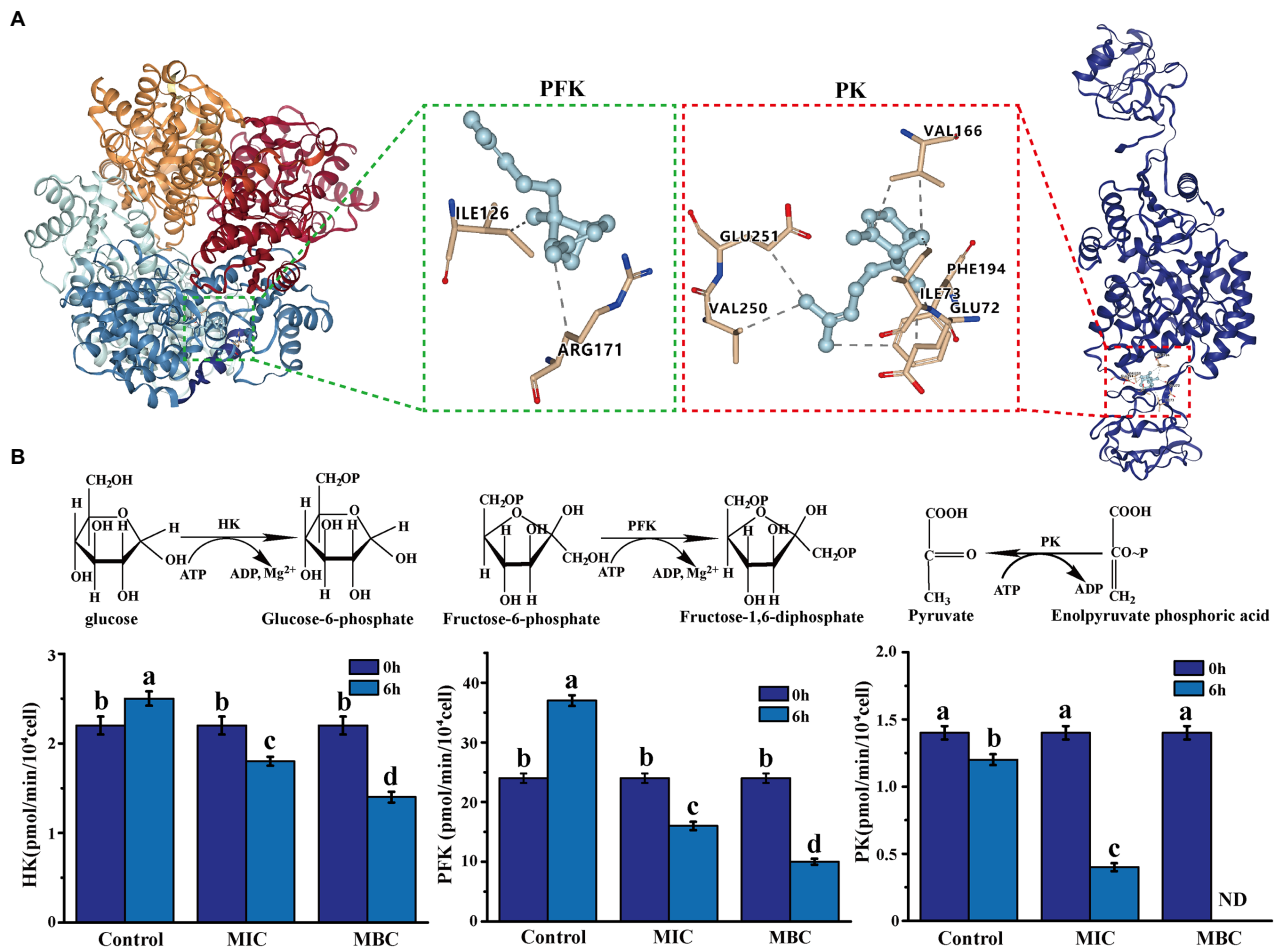
**(A)** The molecular docking of α-bergamotene and the key enzymes [phosphofructokinase (PFK), pyruvate kinase (PK)] of the EMP pathway. **(B)** The effect of BEO on key enzyme activities of EMP pathway. Different letters in the figure indicate significant difference ( *p* <0.05).

Molecular docking is a powerful tool widely used in drug discovery to help understand the molecular recognition mechanism between small and large molecules (Pinzi and Rastelli, 2019). Therefore, molecular docking technology was used to predict the mechanism of action between three main components of BEO (linalool, 1,8-cineole, and α-bergamotene) and key enzymes. The molecular docking results showed that α-bergamotene had the best binding force with PFK and PK (Table 3). Figure 6A shows the interaction of receptor and ligand. The α-bergamotene was docked into the combined pocket of PFK and PK. PFK binds to the residues ARG171 and ILE126 of PFK through hydrophobic interactions. Also, PK binds to residues GLU251, VAL250, VAL166, PHE194, GLU72, and ILE73 through hydrophobic interactions. Therefore, BEO interacts with key enzymes through its component α-bergamotene.

**Table 3:** Binding affinity results from molecular docking analysis.

Receptor	Ligand	Grid_Score	Grid_vdw_energy	Grid_es_energy
PFK	Linalool	−32.1636	−30.4149	−1.7487
	Cineole	−29.8033	−27.5048	−2.29844
	Bergamot	−38.1218	−38.0226	−0.09914
PK	Linalool	−38.3045	−37.6555	−0.64902
	Cineole	−33.1909	−34.0818	0.890944
	Bergamot	−44.5843	−44.5889	0.004529

#### The Effect of BEO on the Activity of Key Enzymes in Respiratory Metabolism

The results of molecular docking were verified by measuring the effect of BEO on key enzyme activities. As shown in Figure 6B, after 6 h of incubation, the enzyme activities of the three key enzymes in the control group did not change significantly or increased. At the same time, after the BEO treatment of MIC, the enzyme activities of HK, PFK, and PK decreased by 39.42, 60.44, and 69.64%, respectively. After MBC’s BEO treatment, activities of HK and PFK decreased by 70.21 and 79.87%, and PK activity even reached an undetectable level. Therefore, BEO inhibits the enzyme activity of the key enzyme through the interaction of the component α-bergamotene and the key enzyme, thereby inhibiting the EMP pathway and then affecting the normal physiological metabolism of the bacteria.

Therefore, the effect of BEO can inhibit the respiratory metabolism of *L. monocytogenes* and cause its energy metabolism disorder. This is consistent with the results of the metabonomics study of the inhibitory mechanism of linalool on *L. monocytogenes* by He et al. (2021). The respiratory chain of prokaryotic cells is located on the cell membrane, so the destruction of the respiratory system is consistent with the finding that the cell membrane structure is damaged.

## Conclusion

In this study, spectroscopy technology and molecular docking prediction technology were used to explore the potential antibacterial mechanism of BEO against *L. monocytogenes* from the aspects of cell membrane barrier and respiratory metabolism (the main energy-producing metabolic pathway). The results show that the inhibitory effect of BEO on *L. monocytogenes* has a multi-target effect. Linalool, the main component of BEO, affects the function and physiological activity of the cell membrane by acting on the hydrocarbyl chain of the phospholipid tail of the cell membrane. In addition, molecular docking results revealed that the inhibitory effect of BEO on bacterial respiratory metabolism was achieved through the interaction of α-bergamotene and key enzyme amino acid residues. As a result, it deeply reveals the multi-target antibacterial mechanism of BEO against *L. monocytogenes* and provides theoretical support for it as a potential natural antibacterial agent.

## Data Availability Statement

The original contributions presented in the study are included in the article/Supplementary Material, further inquiries can be directed to the corresponding authors.

## Author Contributions

CL, HC, and LL conceived and designed the experiments in addition to writing the manuscript. CZ and XC conducted the experiments and data analyses. CL, CZ, and XC performed most of the experiments, while HC and LL supervised the execution of the experiments. All authors contributed to the article and approved the submitted version.

## Funding

This research project was financially supported by State Key Laboratory of Utilization of Woody Oil Resource (Grant no. 2019XK 2002), National Natural Science Foundation of China (Grant no. 31972172), Natural Science Foundation of Jiangsu Province (Grant no. BK20201417), Jiangsu Province Research Fund (Grant no. JNHB-131), and Jiangsu University Research Fund (Grant no. 11JDG050).

## Conflict of Interest

The authors declare that the research was conducted in the absence of any commercial or financial relationships that could be construed as a potential conflict of interest.

## Publisher’s Note

All claims expressed in this article are solely those of the authors and do not necessarily represent those of their affiliated organizations, or those of the publisher, the editors and the reviewers. Any product that may be evaluated in this article, or claim that may be made by its manufacturer, is not guaranteed or endorsed by the publisher.

## Footnotes

^1^ https://swissmodel.expasy.org/

^2^ https://www.rcsb.org/

^3^ https://www.sciencedirect.com/topics/medicine-and-dentistry/pubchem

^4^ http://www.yinfotek.com/
