# Supplementary material for: The Interference Mechanism of Basil Essential Oil on the Cell Membrane Barrier and Respiratory Metabolism of Listeria monocytogenes
Source: Front Microbiol. 2022 Apr 1;13:855905. doi: 10.3389/fmicb.2022.855905 (PMC9010862; doi:10.3389/fmicb.2022.855905)
Supplement: Supplementary file 1 [file Data_Sheet_1.DOCX]

Supplementary Material

## Supplementary Figures


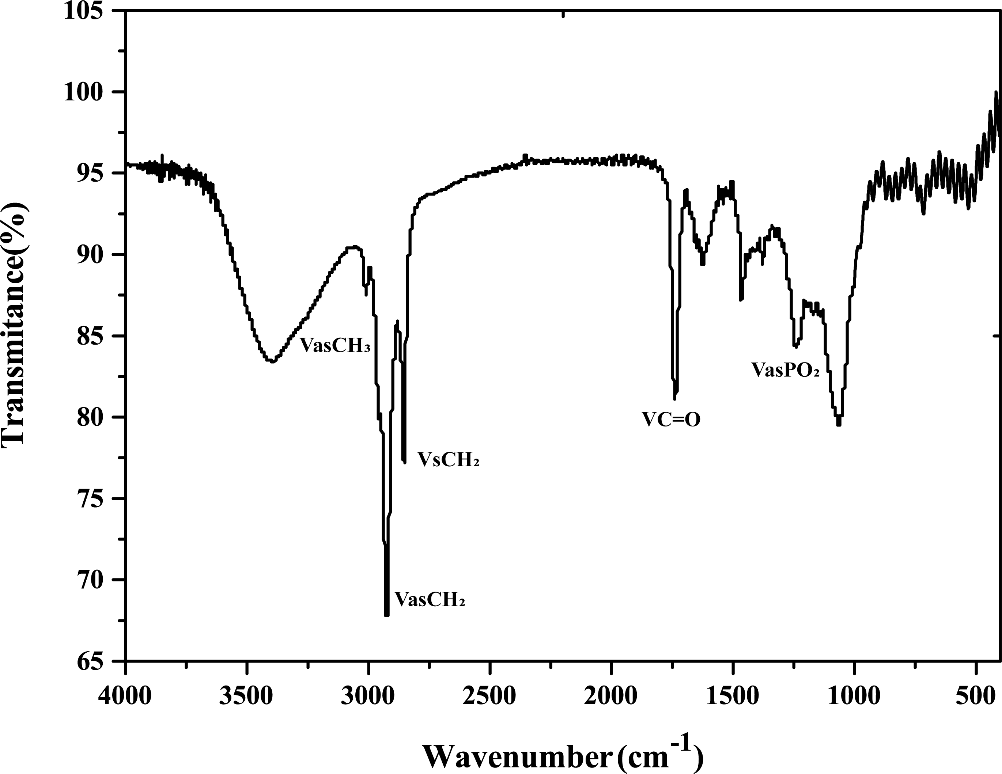


**Supplementary Fig 1.** FTIR Spectra of *L. monocytogenes’* Cell Membrane Phospholipids.


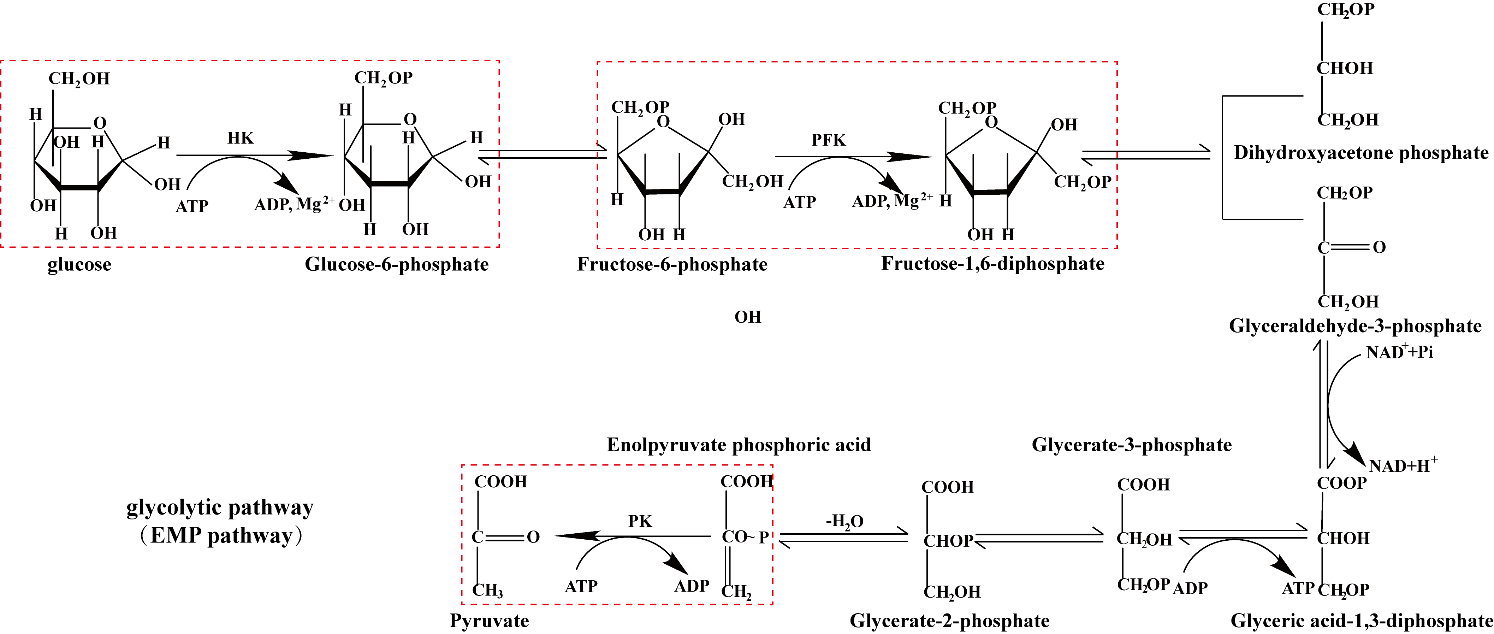


**Supplementary Fig 2.** Schematic diagram of EMP pathway.
